# *Pten* Regulates Development and Lactation in the Mammary Glands of Dairy Cows

**DOI:** 10.1371/journal.pone.0102118

**Published:** 2014-07-10

**Authors:** Zhuoran Wang, Xiaoming Hou, Bo Qu, Jie Wang, Xuejun Gao, Qingzhang Li

**Affiliations:** Key Laboratory of Dairy Science of Education Ministry, Northeast Agricultural University, Harbin, Heilongjiang, China; Wayne State University, United States of America

## Abstract

*Pten* is a tumor suppressor gene regulating many cellular processes, including growth, adhesion, and apoptosis. In the aim of investigating the role of *Pten* during mammary gland development and lactation of dairy cows, we analyzed *Pten* expression levels in the mammary glands of dairy cows by using western blotting, immunohistochemistry, and quantitative polymerase chain reaction (qPCR) assays. Dairy cow mammary epithelial cells (DCMECs) were used to study the function of *Pten in vitro*. We determined concentrations of β-casein, triglyceride, and lactose in the culture medium following *Pten* overexpression and siRNA inhibition. To determine whether *Pten* affected DCMEC viability and proliferation, cells were analyzed by CASY-TT and flow cytometry. Genes involved in lactation-related signaling pathways were detected. *Pten* expression was also assessed by adding prolactin and glucose to cell cultures. When *Pten* was overexpressed, proliferation of DCMECs and concentrations for β-casein, triglyceride, and lactose were significantly decreased. Overexpression of *Pten* down-regulated expression of MAPK, CYCLIN D1, AKT, MTOR, S6K1, STAT5, SREBP1, PPARγ, PRLR, and GLUT1, but up-regulated 4EBP1 in DCMECs. The *Pten* siRNA inhibition experiments revealed results that opposed those from the gene overexpression experiments. Introduction of prolactin (PRL) increased secretion of β-casein, triglyceride, and lactose, but decreased *Pten* expression levels. Introduction of glucose also increased β-casein and triglyceride concentrations, but did not significantly alter *Pten* expression levels. The *Pten* mRNA and protein expression levels were decreased 0.3- and 0.4-fold in mammary glands of lactating cows producing high quality milk (milk protein >3.0%, milk fat >3.5%), compared with those cows producing low quality milk (milk protein <3.0%, milk fat <3.5%). In conclusion, *Pten* functions as an inhibitor during mammary gland development and lactation in dairy cows. It can down-regulate DCMECs secretion of β-casein, triglyceride, and lactose, and plays a critical role in lactation related signaling pathways.

## Introduction

Mammary glands, the lactation organs of the mammals, are regulated by prolactin (PRL) to produce milk. Milk produced during lactation for newborns is generally considered the best nutritional source because it contains optimal ingredients for healthy growth and development [1]. The amino acid content and ratio of components in dairy cow milk, which contain 3.2% milk protein, 4.0% milk fat, and 4.5% lactose, is similar to that necessary for humans, therefore, it is easily absorbed by the human body. An understanding of how the mammary gland is regulated to produce milk is of biomedical and agricultural importance.

There are many transcription factors related to mammary gland development. PRL has a central role in cellular growth, differentiation, secretion, and involution of the mammary gland. PRL-induced activation of Janus kinase 2 (JAK2) and Signal transducer and activator of transcription 5 (STAT5) is required to induce expression of most, possibly all, milk protein genes [2], [3]. PRL also activates Phosphotidylinositol 3 kinase (PI3K) - serine/threonine protein kinase Akt (AKT) cascade, which plays a prominent role in proliferation and survival [4]. AKT can stimulate the translation of milk proteins through its ability to phosphorylate eukaryotic translation initiation factor 4E binding protein 1 (4EBP1), and the AKT-dependent activation of mammalian target of rapamycin (MTOR) stimulates translation through ribosomal protein S6 kinase (S6K1) and eukaryotic translation elongation factor 2. The roles of sterol regulatory element binding protein 1 (SREBP1) and peroxisome proliferator-activated receptor gamma (PPARγ) are to regulate the expression of a number of key lipid metabolism genes [5]. Glucose transporter 1 (GLUT1) is the major glucose transporter in the basal membrane and its expression is regulated by PRL when the demand for glucose during lactose synthesis is amplified [6].

*Pten* (phosphatase and tensinhomolog) is a well-established tumor-suppressor gene, and is one of the most frequently mutated genes in human tumors. Germline mutations of *Pten* can induce cancer-predisposition syndromes [7]–[9]. The PTEN protein is expressed in all tissues in the body, and contains a tensin-like domain along with a phosphatase catalytic domain [10]. *Pten* regulates growth, adhesion, and apoptosis, among many other cell processes. Recent reports on mice with systemic overexpression of *Pten* have expanded our understanding of its physiological functions. *Pten* transgenic mice showed increased energy expenditure, decreased adiposity, improved insulin sensitivity during high-fat feeding or with aging, and extended lifespans. This has led to new mechanistic insights regarding the role of *Pten* in metabolism [11].

*Pten* has an important role in the mammary gland epithelium. It can regulate the growth of mammary cells, and their proliferation and survival by down-regulating several pathways such as PI3K-AKT, Focal adhesion kinase (FAK), and mitogen-activated protein kinases (MAPK) [12]. A recent study showed that the PTEN-AKT pathway is required for the initiation of lactation through the induction of autocrine PRL, providing a direct link between the AKT and STAT5 pathways. The production of autocrine PRL is regulated by the PTEN-PI3K-AKT pathway. Conditional activation of the PI3K-AKT pathway in the mammary glands of virgin mice by either AKT1 expression or *Pten* deletion rapidly induced terminal mammary epithelial differentiation that was accompanied by the synthesis of milk [13]. Mammary gland differentiation was due to PI3K-AKT-dependent synthesis and secretion of autocrine PRL and downstream activation of the prolactin receptor (PRLR)-JAK-STAT5 pathway [14]. It has also been shown that *Pten* overexpression can suppress proliferation and differentiation, and enhance apoptosis of the mouse mammary epithelium; this is accompanied with a significant reduction in milk production [12]. We hypothesized that *Pten* might participate in regulating mammary gland development and lactation in dairy cows.

## Materials and Methods

### Animals and tissue samples

Six healthy multiparous Holstein cows were obtained from the Holstein Cattle Association of Australia. Cows were 100 days postpartum with an average (mean±s.e.) weight of 609±9.08 kg and average parity of 3.1±0.19. They were split into two groups (n = 3 animals per group): the high quality milk (milk yield 30.8±0.76 kg/day, milk protein >3%, milk fat >3.5%), and low quality milk (milk yield 30.6±0.78 kg/day, milk protein <3%, milk fat <3.5%) groups. Prior to the commencement of our study, all cows were healthy and provided a standard feed *ad libitum* comprising 30% roughage and 70% concentrate (Table S1). All diets were formulated using the Cornell−Penn−Miner system (CPM-Dairy, version 3.0.7) to meet the metabolizable energy and protein requirements of cows. Cows were housed in individual tie stalls with continuous access to fresh water, and were milked three times daily. All animals received humane care as outlined in the Guide for the Care and Use of Experimental Animals of the National Institutes of Health. All experimental procedures with animals used in the present study were approved by the Northeast Agricultural University Provincial Experimental Animal Management Committee. All surgeries were conducted with an effort to minimize suffering to animals.

Cows were slaughtered by exsanguination and mammary tissue was aseptically excised 5 cm from the base of the healthy nipple, and 3 cm from the half-line that divides the core of the secretory gland tissue. After removing the connective tissue, the remaining tissue was cut into small blocks with a thickness of 1 cm. Mammary tissue samples were frozen immediately in liquid nitrogen and stored at −80°C for later analysis.

Mammary gland tissue samples were harvested from cows as mentioned above and tissue sections were prepared [15] for immunohistochemistry.

### Dairy cow mammary epithelial cell (DCMEC) cultures

Primary DCMECs were cultured and sub-cultured as previously described [16] in Dulbecco’s modified Eagle’s medium-F12 (Gibco) supplemented with 10% fetal bovine serum (FBS), 5 µg/mL insulin (Sigma, Oakville, ON, Canada), 100 U/mL penicillin and 100 µg/mL streptomycin. For experimental assays, cells in the logarithmic growth phase were cultured in cell flask at 37°C with 5% CO_2_. Purified cells were cultured with serum-free medium for 12 h before further treatment; no supplements were included during serum starvation.

### Generation of pGCMV-*Pten*-IRES-EGFP and transfection

Total RNA was extracted from mammary gland tissue and cDNA was generated using M-MLV reverse transcriptase (TaKaRa). *Pten*-specific primer sequences (sense 5′-GGA ATT CCC GTT CCG AGG ATT ATT C-3′; antisense 5′-GGG GTA CCG TAA AAC AAG ATT GGT CAG G-3′) were used to amplify the desired sequence. After digestion of the PCR products with *Eco*RI and *Kpn*I, the *Pten* gene segment was cloned into pMD18-T (Ambion) to generate pMD18-T-*Pten*. All clones were verified by DNA sequencing. Cloning of the *Pten* gene segment into pGCMV-IRES-EGFP (Ambion) was conducted using a similar method to that for pMD18-T-*Pten*.

DCMECs were transfected with the pGCMV-*Pten*-IRES-EGFP (recombinant plasmid) or pGCMV-IRES-EGFP (empty vector) using Lipofectamine 2000 (LF2000) according to the manufacturer’s recommendations (Invitrogen). Briefly, DCMECs (1×10^6^ cells per well) were plated in 6-well culture plates. For each well, 1 µg of the appropriate plasmid DNA and 2.5 µL of LF2000 was diluted in 200 µL of OPTI-MEMI medium and incubated at room temperature for 20 min to allow for the formation of lipocomplexes; complexes were then added to wells. Cells were incubated with serum- and antibiotic-free medium at 37°C for 36 h. Optimal transfection conditions were screened in advance (Figure S3).

### Transfection of small interfering RNAs (siRNAs)

*Pten* siRNAs and negative scrambled control siRNA were purchased from ShangHai GenePharma. We screened siRNA-*Pten*-a (sense 5′-GGG UAA ACA CAU UCU UCA UTT-3′; antisense 5′-AUG AAG AAU GUG UUU ACC CTT-3′), siRNA-*Pten*-b (sense 5′-CCA GAG GCU AGC AGU UCA ATT-3′; antisense 5′-UUG AAC UGC UAG CCU CUG GTT-3′), and siRNA-*Pten*-c (sense 5′-GCA CAA GAG GCC CUA GAU UTT-3′; antisense 5′-AAU CUA GGG CCU CUU GUG CTT-3′) for highest knockdown efficiency, and chose siRNA-*Pten*-c (Figure S4). The negative scrambled control siRNAs lacked significant sequence homology to any gene (sense 5′-UUC UCC GAA CGU GUC ACG UTT-3′; antisense 5′-ACG UGA CAC GUU CGG AGA ATT-3′). DCMECs were either transfected with siRNA-*Pten*-c (*Pten* siRNA) or negative control using LF2000 according to the manufacturer’s protocol (Invitrogen); untransfected cells were also included as controls. DCMECs were cultured in 6-well plates overnight; for each well to be transfected, 1 µg of siRNA and 2 µL of LF2000 were diluted in 200 µL of OPTI-MEMI medium. The siRNA-LF2000 mixtures were incubated at room temperature for 20 min and then added to well. Cultures were incubated with serum- and antibiotic-free medium at 37°C for 48 h. Optimal transfection conditions were screened in advance (Figure S3).

### Glucose and PRL treatment

DCMECs in the logarithmic growth phase were plated at a concentration of 1.0×10^6^ cells/mL in 6-well culture plates. One group of cells was treated with serum- and antibiotic-free medium as normal control (non-treated group), while three other groups were treated with serum- and antibiotic-free medium with PRL (12 mM) and glucose (20 mM), PRL only, or glucose only. The choice of these concentrations was based on [17] findings in previous work. DCMECs were cultured in a humidified atmosphere at 37°C with 5% CO_2_ for 24 h.

### Quantitative polymerase chain reaction (qPCR) assays

Total RNA from dairy cows mammary gland tissue of high and low quality milk was isolated with ice-cold Trizol solution (Invitrogen Life Technologies, Carlsbad, CA, USA). The quantity and purity of RNA samples were verified by analyzing 5 µL of each sample on a 1% agarose gel and ultraviolet spectrophotometer (Beckman DU800, U.S.A); total RNA integrity was verified using OD_260/280_ ratio and only samples with a ratio greater than 1.8 were used [18]. Reverse transcription system (TaKaRa, Tokyo, Japan) was used to synthesize first-strand cDNA, and qPCR was performed to determine the expression of *Pten* [19]. Reactions were carried out in final volumes of 20 µL using an ABI PRISM 7300 Real-Time PCR System (Applied Biosystems, Foster City, CA, USA). The PCR cycle was as follows: 95°C for 30 s, 40 cycles of 95°C for 5 s and 60°C for 34 s and followed by one cycle at 95°C for 15 s, at 60°C for 1 h and at 95°C for 15 s. Primers were designed with Primer primier 5.0 (PREMIER Biosoft, Palo Alto, CA, USA) and the primer efficiencies in the amplification system were calculated using the standard curve method [20] (Table 1). The qPCR data were analyzed with a 2^−ΔΔCt^ method [21] and normalized using *β-Actin* cDNA as an internal control.

**Table 1:** Oligonucleotide primer sequences used for qPCR assays.

Gene	Forward (F) or reverse (R) Primer	Sequence(5′-3′)	Amplicon size	Primer efficiency
*Pten*	F	CACCTATTCCTCAGCCCTTAT	273 bp	0.99
	R	AACCCTCATTCAGACCTTCAC		
*Mapk*	F	GTCGCCATCAAGAAAATCAGC	309 bp	1.05
	R	GGAAGGTTTGAGGTCACGGT		
*CyclinD1*	F	GGACCGCTTCCTGTCGCT	204 bp	0.89
	R	GCCAGGTTCCACTTGAGTTTGT		
*S6k1*	F	CTGGGTGAAGAATGGAAGGG	101 bp	0.99
	R	CGAACTCTGCCATGGGTCA		
*4Ebp1*	F	TTTGAGATGGACATTTAAAGGGC	101 bp	0.94
	R	CTTGCATAAGGCCTGGCTG		
*Elf5*	F	CACCTATTCCTCAGCCCTTAT	273 bp	0.92
	R	AACCCTCATTCAGACCTTCAC		
*Stat5*	F	GTCCCTTCCCGTGGTTGT	614 bp	1.00
	R	CGGCCTTGAATTTCATGTTG		
*Mtor*	F	ATGCTGTCCCTGGTCCTTATG	178 bp	0.92
	R	GGGTCAGAGAGTGGCCTTCAA		
*Csn2*	F	CCATAACAGCCTCCCAC	111 bp	0.94
	R	GCCATAGCCTCCTTCAC		
*Srebp1*	F	CCAGCTGACAGCTCCATTGA	67 bp	0.97
	R	TGCGCGCCACAAGGA		
*Pparγ*	F	TCAAAGTGGAGCCTGTATC	138 bp	0.95
	R	CATAGTGGAACCCTGACG		
*Glut1*	F	CTTCATCCCAGCCCTGTT	193 bp	1.00
	R	GACCTTCTTCTCCCGCATC		
*Akt*	F	TAAAGAAGGAGGTCATCGTGG	181 bp	0.98
	R	CGGGACAGGTGGAAGAAAA		
*β-Actin*	F	AAGGACCTCTACGCCAACACG	249 bp	0.94
	R	TTTGCGGTGGACGATGGAG		

After overexpression and siRNA inhibition of *Pten*, total RNA from transfected DCMECs was isolated and reverse transcribed into cDNA. The cDNA samples were subjected to qPCR under standard conditions as described above. Data were expressed as fold-changes compared with controls for each experiment. The mRNA expression levels of *Pten, Mapk, Cyclin D1, Akt, Mtor, S6k1, 4Ebp1, Stat5, Elf5, Srebp1, Pparγ, Prlr,* and *Glut1* were assessed.

After incubation with PRL and glucose for 24 h, total RNA from the four DCMEC groups were isolated and reverse-transcribed into cDNA. Reverse transcription products were subjected to qPCR under standard conditions as described above and mRNA expression levels of *Pten* were assessed.

### Western blotting analysis

Proteins were extracted from frozen mammary gland tissue of high and low quality milk samples. Equal amounts of proteins (30 µg) were subjected to SDS-PAGE on polyacrylamide separating gels [19], [22]. Electrophoresed proteins were then transferred to nitrocellulose membrane. After transfer, mambranes were blocked in an isotonic solution containing 5% non-fat dry milk in PBS. Membranes were then incubated with rabbit polyclonal antibodies against PTEN and mouse polyclonal antibodies against β-Actin (Santa Cruz Biotechnology Inc., Santa Cruz, CA, USA) as primary antibodies. Depending on the origin of the primary antibody, either goat anti-rabbit or anti-mouse HRP conjugated IgG (Zhongshan-Bio, Beijing, China) was used for detection using ECL system (ApplyGEN, Beijing, China). We used β-Actin as a loading control.

After overexpression and siRNA inhibition of *Pten*, proteins were extracted from transfected DCMECs. Equal amounts of proteins (30 µg) were prepared for western blotting as described above. We used polyclonal rabbit antibodies against p38 MAPK, phospho-p38 MAPK, AKT, phospho-AKT (Ser473), MTOR, phospho-MTOR, S6K1, phospho-S6K1 (Thr421/Ser424), STAT5, phospho-STAT5 (Cell Signaling Technology, Beverly, MA, USA), PPARγ and PRLR (Abcam Technology, Cambridge, MA, USA), PTEN, CYCLIN D1, and GLUT1 (Santa Cruz Biotechnology Inc.), as well as polyclonal goat antibodies against 4EBP1 and ELF5, and polyclonal mouse antibodies against SREBP1 and β-Actin (Santa Cruz Biotechnology Inc.) as primary antibodies. Goat anti-rabbit, goat anti-mouse, and donkey anti-goat HRP conjugated IgG (Zhongshan-Bio) were used as secondary antibodies for detection using ECL (ApplyGEN), and β-Actin was used as a loading control.

After exposure to PRL and glucose for 24 h, proteins were extracted from cultured DCMECs. Equal amounts of proteins (30 µg) were prepared for western blotting as described above. We used a polyclonal rabbit antibody against PTEN and a polyclonal mouse antibody against β-Actin (Santa Cruz Biotechnology Inc.) as primary antibodies. Goat anti-rabbit and goat anti-mouse HRP conjugated IgG (Zhongshan-Bio) were used as secondary antibodies for detection using ECL (ApplyGEN), and β-Actin was used as a loading control.

### Immunohistochemistry

Tissues dissected from the nipples of cows were fixed in 4% paraformaldehyde and embedded in paraffin. Sections (5-µm thickness) were deparaffinized with xylene, rehydrated, and treated with 3% H_2_O_2_ to quench the endogenous peroxidase activity. Antigen retrieval was performed by microwaving sections in citrate buffer solution; sections were then blocked by treating slides with 1% fish skin gelatin, followed by incubation with an anti-PTEN antibody (1∶100; Santa Cruz Biotechnology Inc.). After washing, slides were treated with a FITC-conjugated goat anti-rabbit IgG (1∶50; Santa Cruz Biotechnology Inc.), followed by incubation in 1 µg/mL DAPI for 10 min, and finally sections were mounted on slides with Antifade Mounting Medium (Beyotime, China). Between each step sections were washed three times in deionized water [22]. Images were captured using a laser-scanning confocal microscope (Leica TCS SP2 AOBS, Germany). Image-Pro Plus (IPP) 6.0 software (Media Cybernetics Inc., Bethesda, MD, USA) was used for quantifying the mean density of PTEN signals, and sections (n = 3 for each group) of the implantation site were used to quantify PTEN protein levels [23].

### Cells viability assays

DCMECs were transfected with pGCMV-*Pten*-IRES-EGFP, pGCMV- IRES-EGFP, *Pten* siRNA or negative control siRNA. The cell viability was determined using the CASY-TT Analyzer System (Schärfe System GmbH, Reutlingen, Germany) according to the manufacturer’s instructions. After calibration with dead and viable DCMECs cursor positions were set at 11.75 to 50.00 µm (evaluation cursor) and 7.63−50.00 µm (standardization cursor). After trypsinizing, aliquots (100 µL) of cells were diluted with CASY electrolyte solution (1∶100) and analyzed in triplicate using CASY-TT [24].

### Cell cycle analysis

Following transfection, DCMECs were harvested by trypsin digestion and centrifugation. Cells were then washed with cold phosphate-buffered saline (PBS) fixed in 70% (v/v) ethanol at 4°C overnight, washed again, and incubated with 50 µg/mL propidium iodide and 2 µl/mL TritonX-100 for 20 min at room temperature in the dark. Cells were resuspended in 500 µl of PBS and subjected to flow cytometry on a Cytomics FC500 flow cytometer. Percentages of cells within each phase of the cell cycle were determined using ModFit LT 3.2 software (Verity Software House, USA) [25], [26].

### Changes in β-casein, triglyceride and lactose secretion

Following transfection, DCMECs were incubated with serum- and antibiotic-free medium for 36 h (*Pten* overexpression) or 48 h (siRNA inhibition of *Pten*). Culture medium were then collected using a glass dropper and transferred to one of the following detection kits (all used according to manufacturer’s instructions): ELISA Kit for Casein Beta (CSN2; New England Biolabs Inc.,Beverly, MA, USA); Triglyceride (TG) GPO-POD assay kit (Applygen Tech Inc.) and Lactose & D-galactose (Rapid) assay kit (Megazyme, Bray Business Park, Bray, Ireland). After DCMECs were treated with PRL and glucose for 24 h as described previously, culture medium were collected for detection using the a forementioned kits.

### Statistical analysis

Data were analyzed using SPSS 13.0 (SPSS, Chhicago, IL, USA). All the experimental data were expressed as means ± standard deviation (SD). All data were tested for a normal distribution using the Shapiro-Wilk test and for homogeneity of variances by Levene’s test. Since all data were normally distributed, when they had similar variances, Student’s *t*-test (comparisons of the two groups of dairy cow mammary gland tissues) or one-way analysis of variance (ANOVA) (experiments involving *Pten* overexpression and siRNA inhibition; *Pten* expression levels, triglyceride and lactose secretion after PRL and glucose treatment) were conducted to compare means among all measured variables. When ANOVA results were significant, multiple comparisons of means were carried out with Tukey HSD post-hoc analysis. When the data did not have similar variances (subjects of β-casein secretion after PRL and glucose treatment), the non-parameter Kruskal−Wallis test for comparing the median was applied, along with the Mann−Whitney test for multiple comparisons among the different groups. A *P*-value less than 0.05 was considered statistically significant. All the experiments were repeated at least for three times.

## Results

### *Pten* mRNA and PTEN protein expression in tissue

**Figure 1:**
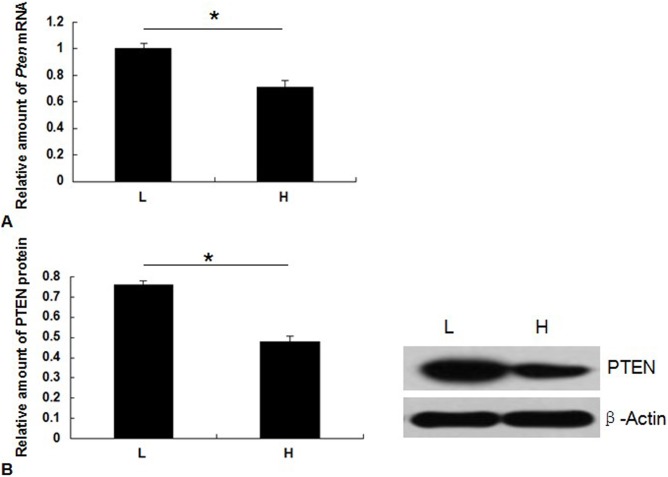
PTEN mRNA and protein expression levels in dairy cow mammary tissues during lactation. (A) The mRNA expression levels of *Pten* as determined by qPCR. (B) Western blotting detection of PTEN; L, tissue from cows with low quality milk; H, tissue from cows with high quality milk. *P<0.05.

Quantitative analysis of *Pten* mRNA expression in dairy cow mammary tissues showed that *Pten* mRNA levels were significantly decreased during lactation for cows producing high quality milk compared with those producing low quality milk (*P*<0.05; Figure 1). Consistent with the mRNA expression levels, higher PTEN protein expression levels were observed during lactation in cows with low quality milk compared with those that produced high quality milk (*P*<0.05; Figure 1). Our immunohistochemistry results supported our mRNA and protein expression data. PTEN immunostaining was more evident in cows with low quality milk than those with high quality milk (*P*<0.05). The difference in mean optical density of the nuclei for the groups was insignificant (Figure S1).

### *Pten* regulates DCMEC functions

For cultured DCMECs, cytokeratin-18 was used as a marker to ensure that we obtained pure mammary epithelial cells [27] (Figure S2). Intracellular localization of PTEN was observed in DCMECs (Figure S1). Before *Pten* transfection, an equivalent number of cells were seeded in cell culture plates. Recombinant plasmids were generated, and verified by DNA sequencing. The best transfection conditions and efficiency were optimized for *Pten* overexpression and siRNA inhibition in advance (Figure S4).

#### *Pten* regulates viability of DCMECs

**Figure 2:**
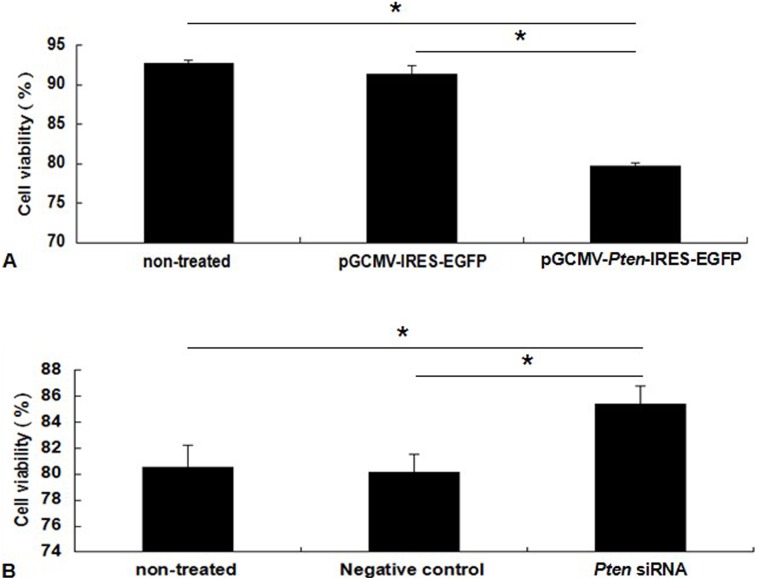
*Pten* regulates the viability of DCMECs after transfection. (A) DCMECs were transfected with *Pten* recombinant plasmid or empty vector as a control. Cell viability of DCMECs was determined at 36 h post-transfection. (B) DCMECs were transfected with *Pten* siRNA or negative control. Cell viability of DCMECs at 48 h post-transfection was determined. Values are presented as the mean ± SD, *P<0.05.

Using the CASY-TT Analyser System we discovered that cell viability in *Pten* overexpression and siRNA inhibition groups relative to their respective negative control groups was reduced by 12.76% (*P*<0.05) and increased by 6.61% (*P*<0.05), respectively. These findings suggested *Pten* inhibited cells proliferation and decreased cell viability (Figures 2 and S4).

#### *Pten* regulates the DCMEC cell cycle

We examined cell cycle distribution in DCMECs by using flow cytometry (Tables 2 and 3). Upon *Pten* recombinant plasmid transfection, the cells had a marked increase in G_0_/G_1_ cell population and the percentage of cells in the S and G_2_/M phase were significantly decreased (*P*<0.05) compared with those in the control groups. Following *Pten* siRNA inhibition, the opposite results were seen compared with those for the *Pten* recombinant plasmid.

**Table 2:** *Pten* overexpression regulates the cell cycle in DCMECs (%).

	Cell cycle		
Groups	G_0_/G_1_	S	G_2_/M
non-treated	60.31±0.45^b^	31.39±1.82^a^	8.30±0.90^a^
pGCMV-IRES-EGFP	61.65±0.45^b^	30.35±1.87^a^	8.00±0.90^a^
pGCMV-*Pten*-IRES-EGFP	67.06±0.48^a^	26.09±1.84^b^	6.85±0.97^b^

Note: Values are presented as the mean ± SD, different superscript letters indicate significantly different values in column data, *P*<0.05.

**Table 3:** *Pten* siRNA inhibition regulates the cell cycle in DCMECs (%).

	Cell cycle		
Groups	G_0_/G_1_	S	G_2_/M
non-treated	58.13±1.45^a^	39.26±1.14^b^	2.60±0.92^b^
negative control	57.69±0.64^a^	39.65±1.26^b^	2.66±0.92^b^
*Pten* siRNA	52.60±0.84^b^	43.25±1.68^a^	4.16±0.95^a^

Note: Values are presented as the mean ± SD, different superscript letters indicate significantly different values in column data, *P*<0.05.

#### *Pten* regulates β-casein, triglyceride and lactose secretion in DCMECs

**Figure 3:**
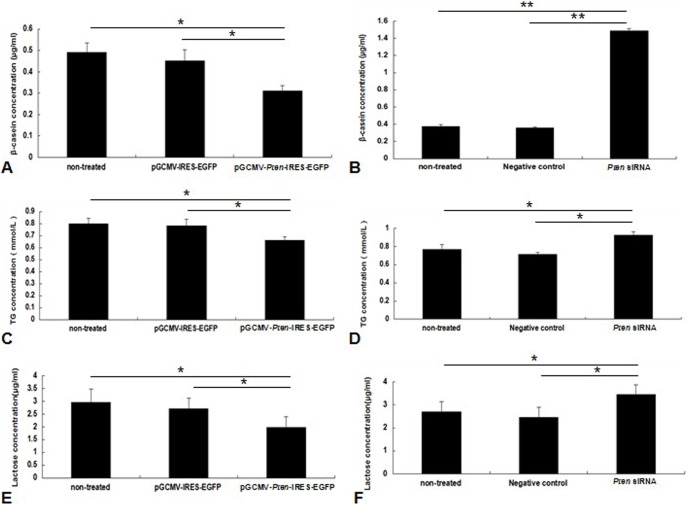
*Pten* regulates mammary epithelial cell secretion of β-casein, triglyceride, and lactose. (A) *Pten* overexpression down-regulates β-casein secretion. (B) *Pten* siRNA inhibition up-regulates β-casein secretion. (C) *Pten* overexpression down-regulates triglyceride secretion. (D) *Pten* siRNA inhibition up-regulates triglyceride secretion. (E) *Pten* overexpression down-regulates lactose secretion. (F) *Pten* siRNA inhibition up-regulates lactose secretion. *P<0.05, **P<0.01.

In comparison with the controls, secretion of β-casein, triglyceride and lactose were significantly suppressed by the overexpression of *Pten* (*P*<0.05; Figure 3); Inhibition of *Pten* with siRNAs resulted in opposing findings (*P*<0.05; Figure 3).

#### *Pten* regulates expression of lactation-related pathway genes

**Figure 4:**
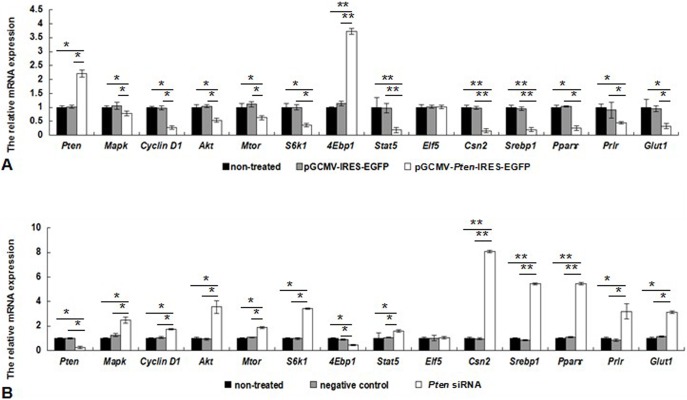
Expression of mRNAs in DCMECs following *Pten* overexpression and siRNA inhibition. (A) DCMECs were transfected with *Pten* recombinant plasmid for 36 h and mRNA levels of *Pten, Mapk, Cyclin D1, Akt, Mtor, S6k1, Stat5, Csn2, Srebp1, Pparγ, Prlr,* and *Glut1* were assessed. (B) Following transfection with *Pten* siRNA, mRNA levels of *Pten, Mapk, Cyclin D1, Akt, Mtor, S6k1, Stat5, Csn2, Srebp1, Pparγ, Prlr,* and *Glut1* were determined at 48 h post-transfection. Expression was calculated relative to *β-Actin* expression. *P<0.05, **P<0.01.

**Figure 5:**
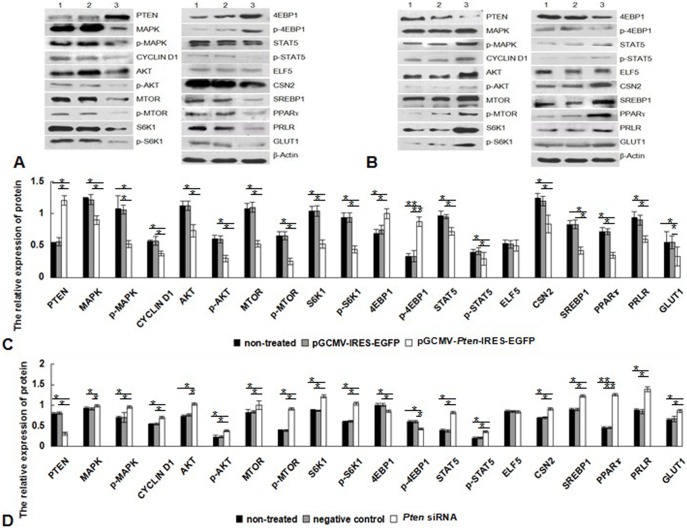
Expression and activation of key lactation-related pathway proteins following *Pten* overexpression and siRNA inhibition. (A) Western blotting detection of PTEN, MAPK CYCLIN D1, AKT, MTOR, S6K1, STAT5, CSN2, SREBP1, PPARγ, PRLR, and GLUT1 after *Pten* overexpression. Lane 1, non-treated group, DCMECs were non-transfected and cultured for 36 h; 2, empty vector group, DCMECs were transfected with pGCMV-IRES-EGFP plasmid for 36 h; and 3, *Pten* overexpression group, DCMECs were transfected with pGCMV-*Pten*-IRES-EGFP recombinant plasmid for 36 h. (B) Western blotting detection of PTEN, MAPK CYCLIN D1, AKT, MTOR, S6K1, STAT5, CSN2, SREBP1, PPARγ, PRLR, and GLUT1 after treatment with *Pten* siRNA. Lane 1, non-treated group, DCMECs were non-transfected and cultured for 48 h; 2, negative control group, DCMECs were transfected with negative control interference segment for 48 h; 3, *Pten* siRNA inhibition group, DCMECs were transfected with siRNA *Pten* for 48 h. (C) Western blotting detection of PTEN, MAPK CYCLIN D1, AKT, MTOR, S6K1, STAT5, CSN2, SREBP1, PPARγ, PRLR, and GLUT1 after *Pten* overexpression for 36 h. (D) Western blotting detection of PTEN, MAPK CYCLIN D1, AKT, MTOR, S6K1, STAT5, CSN2, SREBP1, PPARγ, PRLR, and GLUT1 after treatment with *Pten* siRNA for 48 h. Expression is shown relative to *β-Actin* levels. *P<0.05, **P<0.01.

While there were no significant changes in expression levels of genes between the two control groups (*P*>0.05), the mRNA levels of *Pten* in DCMECs transfected with the *Pten* recombinant plasmid for 36 h were increased 2-fold in comparison with those for control groups (*P*<0.05). The mRNA levels of *Pten* were reduced 4-fold 48 h after transfection with *Pten* siRNA (*P*<0.05). Overexpression of *Pten* also resulted in decreased expression levels of *Mapk, Cyclin D1, Akt, Mtor, S6k1, Stat5, Csn2, Srebp1, Pparγ, Prlr,* and *Glut1* compared with the empty vector and non-treated group (*P*<0.05). Expression of *4Ebp1* mRNA was significantly higher in the *Pten* overexpression group than in the control groups (*P*<0.05; Figure 4 A). Treatment with *Pten* siRNA revealed contrasting results (Figure 4 B). There were no changes in *Elf5* expression levels in the three groups after *Pten* overexpression and siRNA inhibition (*P*>0.05). Similar results were observed at protein level by western blotting analysis (Figure 5). Overexpression of *Pten* up-regulated the expression of 4EBP1, but down-regulated expression of MAPK, CYCLIN D1, AKT, MTOR, S6K1, STAT5, CSN2, SREBP1, PPARγ, PRLR, and GLUT1, treatment with *Pten* siRNA revealed contrasting results, and both *Pten* overexpression and siRNA inhibition showed no significant effects on ELF5.

### β-casein, triglyceride and lactose secretion by DCMECs after PRL and glucose treatment

**Figure 6:**
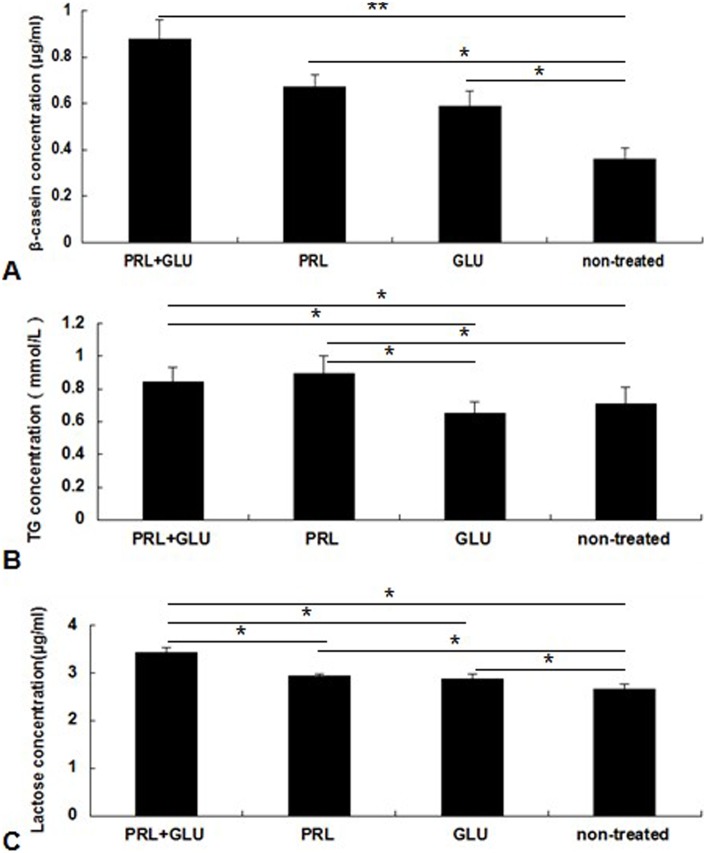
PRL and glucose regulates DCMEC secretion of β-casein, triglyceride and lactose. (A) PRL and glucose regulate β-casein secretion. (B) PRL and glucose regulate triglyceride secretion. (C) PRL and glucose regulate lactose secretion. PRL+GLU, 12 mM PRL and 20 mM glucose for 24 h; PRL, 12 mM PRL for 24 h; GLU, 20 mM glucose for 24 h; non-treated, serum-free medium without supplements for 24 h. Values are presented as the mean ± SD, *P<0.05, **P<0.01.

We analyzed levels of β-casein, triglyceride and lactose in the culture medium of DCMECs (Figure 6). Following treatment with PRL, glucose, or both, the concentration of β-casein was significantly increased compared with those in the non-treated group (*P*<0.05). There were no significant differences among the three treated groups (*P*>0.05). The triglyceride content increased significantly after treatment with PRL and glucose, and PRL only (*P*<0.05). As for lactose, after treatment with either PRL or glucose, the concentration of lactose increased. The lactose level in the group treated with both PRL and glucose was significantly higher than those in the other three groups (*P*<0.05).

### PRL and glucose regulate expression of PTEN mRNA and protein

**Figure 7:**
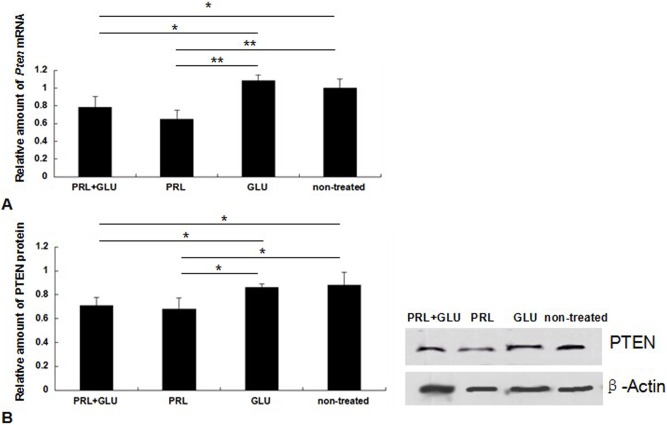
PRL and glucose influence on *Pten* expression. (A) Analysis of *Pten* mRNA expression levels by qPCR. (B) Western blotting detection of PTEN. PRL+GLU, 12 mM PRL and 20 mM glucose for 24 h; PRL, 12 mM PRL for 24 h; GLU, 20 mM glucose for 24 h; non-treated, serum-free medium without supplements for 24 h. Expression is shown relative to *β-Actin* expression. *P<0.05, **P<0.01.

QPCR analysis showed that compared with non-treated group, mRNA levels of *Pten* exhibited a notable down-regulated expression in DCMECs after incubation with PRL alone or PRL in combination with glucose (*P*<0.05) (Figure 7A). Cells incubated with glucose alone showed no significant differences in mRNA expression levels compared with those for the non-treated group. Similar results were observed at protein level by western blotting analysis (Figure 7B). These data indicate that *Pten* expression in DCMECs can be down-regulated by PRL but not by glucose.

## Discussion

The mammary gland represents a unique tissue that undergoes cycles of cell proliferation, differentiation, and apoptosis. *Pten* has the ability to inhibit cellular proliferation, differentiation, and the promotion of apoptosis; it also regulates other biological processes, such as cell migration, invasion, and neoplastic transformation of cells [13]. To explore the role of *Pten* in the development of bovine mammary glands, expression levels of *Pten* in milk of varying quality produced during lactation were investigated using qPCR, western blotting, and immunohistochemistry. *Pten* expression was much lower in high quality milk than in low quality milk, suggesting that *Pten* suppresses the secretion of β-casein, triglyceride, and lactose in mammary epithelial cells.

It is known that the quantity and viability of mammary epithelial cells are associated with milk production [28]. Most mammary epithelial cells are secretory cells that undergo functional differentiation during pregnancy to produce milk. An increased number of mammary epithelial cells and enhanced cell viability will contribute to lactation, however, the mechanisms responsible for variations in the activity and number of mammary cells during lactation in ruminants remain poorly understood [29], [30]. Our CASY-TT results showed that overexpression of *Pten* decreased the viability of mammary epithelial cells. In contrast, transfection of *Pten* siRNA increased the viability of mammary epithelial cells, suggesting that *Pten* could down-regulate DCMEC viability. In the present study, the signaling pathway through which *Pten* regulated cell viability was not clearly identified. We speculate that *Pten* is a novel regulator that inhibits the viability of DCMECs.

The mammary gland is unique in its capability to undergo cycles of cellular proliferation, differentiation, and apoptosis during adult life. Studies have shown that *Pten* can inhibit anchorage-independent growth in the nucleus and promote stagnation of the G_1_ phase [24]. Our flow cytometry results showed that the number of cells in the G_0_/G_1_ phase increased significantly after *Pten* overexpression, whereas the number of cells in the S and G_2_/M phase were decreased. The opposite observations were seen when *Pten* was inhibited. A recent study showed that *Pten* appears to function as a crucial inhibitor of glioblastoma stem cells (GSCs) and causes retardation in growth and senescence [31]. This is in accordance with our results of the viability and proliferation studies. From these results, we suppose that changes in *Pten* activity, through overexpression or siRNA inhibition, can alter the sensitivity of cells to apoptosis, and change the proliferation abilities of DCMECs.

The major milk protein β-casein is also an indicator of lactation ability for mammary epithelial cells [23]. In cow milk, approximately 82% of milk protein is casein (αs1-, αs2-, β- and κ-casein); among them β-casein makes up the greatest proportion with a relatively constant proportion and structure. Cell proliferation determines the rate of cell-cell attachment at distinct stages of mammary gland development, resulting in obvious changes in the expression levels of β-casein, which is a hallmark of mammary gland differentiation [32]. Triglyceride and lactose are also major components of milk. Overexpression of the *Pten* gene resulted in a significant reduction in β-casein, triglyceride and lactose levels. When *Pten* expression was inhibited, β-casein, triglyceride, and lactose secretion were significantly increased. These results indicate that *Pten* regulates the lactation ability of DCMECs. It is difficult to imagine that this can be achieved by simply regulating a certain target gene; instead, we posit that several signaling cascades are involved and that they are combined at the genome level.

The activation of *Pten* is involved in cell survival through downstream MAPK and PI3K-AKT signaling cascades. MAPK-mediated growth signaling pathway can oppose the effects of *Pten* overexpression on proliferation or migration in different cell lines [33]. Our results also showed a correlation between MAPK and PTEN in DCMECs, as MAPK expression declined with *Pten* overexpression. A recent study showed that *Mapk* increased milk protein synthesis through the STAT5 and MTOR pathways, providing new insights into the mechanisms of milk protein synthesis [34]. AKT activation stimulates cell cycle progression, survival, metabolism and migration through phosphorylation of many physiological substrates [35]. It was also shown that AKT is up-regulated at both the mRNA and protein levels during lactation [6], indicating that it may play an important role in mammary gland. It was reported that constitutively active forms of PI3K and AKT could rescue PTEN-induced suppression of cell proliferation. Downstream genes of *Akt* are also involved in a series of processes regulating the synthesis of protein and fat and glycometabolism, suggesting that an AKT-mediated signaling pathway plays a major role in PTEN-sensitive cell proliferation, cell survival, and anabolic metabolism [36]. It was also verified in this study, the mRNA and protein levels of *Akt* were down-regulated by *Pten*, and expression of most downstream genes of *Akt* which related to lactation were influenced by *Pten* overexpression and siRNA inhibition, indicating *Pten* may take part in synthesis of milk protein, fat and lactose as well.

Inactivation of *Pten* could lead to promotion of cell cycle processes by AKT phosphorylation. This in turn maintains the steady expression of CYCLIN D1; overexpression of *Pten* down-regulates CYCLIN D1 [37]. We observed a reduction in AKT and CYCLIN D1 levels in the DCMECs where *Pten* was overexpressed and opposing results when *Pten* was inhibited, consistent with previous studies.

Among the numerous targets of the AKT kinases, MTOR is especially relevant for lactation, and is involved in numerous anabolic processes such as cell growth and protein synthesis [11], [38], [39]. MTOR has two downstream targets: the p70 S6K1, which activity is up-regulated by MTOR and is known to play an important role in cell proliferation and cell cycle progression [40], and 4EBP1, which is negatively regulated by MTOR [41]. The transcription of milk protein genes could be enhanced by AKT1 acting on their substrates, such as MTOR/S6 kinase and MTOR/4EBP1 [42]. It is therefore plausible that *Pten* overexpression, by blocking the action of these effectors downstream of the PI3K-AKT pathway, recapitulates the metabolic change effects observed during MTOR, 4EBP1, and S6K1 deficiency.

PRL is a polypeptide hormone synthesized and released from the anterior pituitary gland that regulates lactation, reproduction, metabolism, immune responses, and electrolyte balance. When secreted into the circulatory system, pituitary PRL binds to the PRLR and activates JAK2-STAT5 signaling pathway, which is required to induce expression of most, or possibly all, of the milk protein genes. The *Stat5* gene is an important regulatory factor in milk protein synthesis. It was previously shown that there was at least one STAT5 binding site in the β-casein promoter [43]. Further work has shown that the differentiation of mammary cells occurred because of PI3K-AKT-dependent synthesis and secretion of autocrine PRL and downstream activation of the PRLR-JAK-STAT5 pathway [44]. Expression of STAT5 and β-casein gene (*Csn2*) was down-regulated by *Pten* at both the mRNA and protein level, indicating that *Pten* regulates STAT5 signaling in the mammary gland. This is accompanied by PI3K-AKT signaling cascades mediating distinct aspects of the production of the three major components of milk: lactose, lipids, and milk proteins [12]. It has also been implied that ELF5 acts upstream of STAT5 signaling, with ELF5 found to bind to the STAT5 promoter [45]. Recent advances suggest that ELF5 is an important mediator of PRL. Forced expression of the ELF5 transcription factor can restore lactation in mice that fail to lactate because of the loss of alleles encoding PRLR [46]. In our current study, expression of ELF5 was not significantly affected by overexpression or siRNA inhibition of *Pten*, indicating that the impact of *Pten* expression on PRLR-JAK-STAT5 pathway is not mediated by ELF5. GLUT1 is involved in the main system for glucose transport in rat, mouse and cow mammary epithelial cells [43]. It was recently shown that the absence of AKT1 specifically resulted in a decrease of GLUT1 that was associated with the basolateral surface of mammary epithelial cells during lactation [7]–[9]. A similar result was observed in our present study, with both AKT and GLUT1 reduced with *Pten* overexpression. Treatment of lactating rats with bromocriptine to inhibit production of PRL by the pituitary gland caused a 37% decrease in GLUT1 levels. Thus, GLUT1 is the major glucose transporter in the basal membrane and its expression is regulated by PRL. In the present study, PRLR and GLUT1 were down-regulated by the expression of *Pten*, indicating that *Pten* has an impact on PRL-induced lactose synthesis.

The PPAR transcription factors are parts of the ligand-activated nuclear hormone receptor superfamily. In the present study, PPARγ was shown to be down-regulated by *Pten*. The STAT5a promoter was recently shown to contain numerous PPAR-responsive elements that mediate the PPARγ regulatory activity of *Stat5a* gene expression in rat mammary cells [47]. *Stat5* is shown to function in adipocyte development, adipocyte differentiation, and lipid accumulation by regulating PPARγ [48]. It is speculated that the expression of *Pten* down-regulates STAT5 and PPARγ to inhibit milk protein and fat synthesis.

Our results provide evidence that decreased *Pten* expression caused increased SREBP1 expression in DCMECs. The SREBP1 peptide is a member of the basic helix-loop-helix transcription factor family, capable of activating the transcription of genes for the synthesis of fatty acids [6]. SREBPs were discovered to be the major nuclear transcription factors that contribute to the regulation of lipid synthesis and secretion. They are able to activate the transcription of genes encoding enzymes such as *Fas, Acs, Scd, Hmgcr*, and subsequently stimulating lipid synthesis and secretion [34]. Thus, *Pten* plays an important role in lipid deposition. A potential role for SREBP1 regulation by AKT was revealed in a study that demonstrated activation of SREBP1 in human retinal pigment epithelial cells expressing activated AKT [49]. Recent reports indicate that activation of AKT is involved in the transport of the SREBP cleavage-activating protein (SCAP)/SREBP complex from the endoplasmic reticulum to the Golgi [50], which is a major step in SREBP activation. AKT-dependent induction of fatty acid synthase requires the presence of SREBPs because induction of gene transcription is blocked by dominant negative mutants of SREBPs or siRNAs directed against SREBP1a and SREBP1c [6]. Further studies are needed to determine the exact genes downstream of SREBP1 that are involved in the PTEN-PI3K-AKT mediated pathway.

PRL acts through its receptor (PRLR) via both endocrine and local paracrine/autocrine pathways to regulate reproduction and lactation [51]. In the lactating mammary gland, PRL increases the production of milk proteins, lactose, and lipids. The additive of PRL induced a decrease in the level of Nuclear Factor1-C2 proteins at lactation, which accomplish initiation of milk gene transcription [52]. During lactation, PRL enhances mammary production of lipids by coordinating the activities of key enzymes [53]. This is also consistent with the findings of our present study where triglyceride content was significantly increased following PRL incubation. We also confirmed that PRL increases the synthesis of β-casein and lactose. Given its osmotic properties, lactose is also the main regulator of milk volume. According to a previous study, DCMECs were cultured with glucose, the results showed that contents of lactose and cell viability rose obviously, results also indicated that glucose could up-regulate expression of *Stat5* gene and the lactation ability of DCMECs [54]. Based on the current literature, increasing glucose availability might partially stimulate lactose synthesis by altering the expression of beta1, 4-galactosyltransferase, thereby increasing milk yield [16]. In the present study, our findings revealed that the content of β-casein and lactose increased after DCMECs were incubated with glucose, but triglyceride concentrations did not change significantly. This could be related to high concentrations of glucose, which decreases expression levels of genes involved in milk fat synthesis. Same result was also observed in previous research where duodenal glucose infusions reduced milk fat production because of a decrease in lipoprotein lipase activity and intramammary esterification [17]. Results of a previous study showed that overexpression of *Prl2* in HEK293 cells leads to a 40% decrease in *Pten*, whereas deletion of *Prl2* gave rise to a 1.7-fold increase in *Pten* in *Prl2*-deficient placentas, providing the first evidence that PRL2 has the ability to negatively regulate *Pten* [44]. In our study, the expression of *Pten* was significantly decreased at both the mRNA and protein levels after incubation with PRL, thereby demonstrating that *Pten* can be down-regulated by PRL in DCMECs. However, the addition of glucose showed no significant influence, and even caused a slight decrease in *Pten* expression in our study, indicating that the addition of glucose likely has no significant effect on *Pten* expression in DCMECs.

## Conclusion

In summary, we showed that *Pten* is specifically involved in dairy cow mammary gland development, and regulates DCMEC viability, proliferation ability, and the cell cycle along with β-casein, triglyceride, and lactose secretion. *Pten* targets and regulates the PI3K-AKT pathway, which in turn regulates other lactation-related signaling genes. The addition of PRL to culture medium resulted in a decrease in DCMEC *Pten* expression levels. We have gained new insights into the role of *Pten* in the dairy cow mammary gland, and the mechanism by which *Pten* regulates DCMEC growth and milk secretion. Ongoing efforts are required to understand the exact mechanism by which *Pten* regulates PRL-induced lactation.

## Supporting Information

Figure S1 **Localization of PTEN in dairy cow mammary tissues and cells.** (A) Confocal microscopy images showing localization of PTEN in dairy cow mammary tissues. H, tissue from cows with high quality milk; L, tissue from cows with low quality milk. (a) PTEN, (b) nuclear staining with propidium iodide (PI), (c) merged image of (a) and (b). The mean optical density of PTEN protein expression in different mammary tissues from high or low quality milk producing and lactating dairy cows (n = 3 in each group) are shown in the table below. Each value is presented as the mean ± SD, different superscript letters indicate significantly different values in line data, *P*<0.05. (B) Localization of PTEN in DCMECs (200×). (1) PTEN; (2) nuclear staining with PI; and (3) merged images of (1) and (2).
(TIF)

Figure S2 **Cultured dairy cow mammary gland epithelial cells.** (A) Collagenoblast (200×). (B) Collagenoblast and DCMECs (200×). (C) Purified DCMECs (200×). (D) Serial cultures of DCMECs (200×). (E) Cytokeratin 18 staining of mammary epithelial cells. (F) Cytokeratin 18 staining of fibroblast cells. Nuclei were stained with PI.
(TIF)

Figure S3 **Optimization of transfection conditions**. (A) Relative mRNA levels of DCMECs following transfection with *Pten* recombinant plasmid were determined using qPCR. Expression of *Pten* mRNA levels peaked at 36 h. Expression was calculated relative to expression levels at 0 h. *P<0.05, **P<0.01. (B) Screening of siRNA efficiency and incubation times. Relative mRNA levels in DCMECs transfected with various siRNAs (siRNA-*Pten*-a, siRNA-*Pten*-b and siRNA-*Pten*-c) at different time points as determined by qPCR. Expression was determined relative to expression levels at 0 h. *P<0.05, **P<0.01. (C) Transfection efficiency as determined by laser confocal microscopy (200×). DCMECs were transfected with the *Pten* recombinant plasmid for 36 h. Nuclei were stained with DAPI, and PTEN was detected by visualizing green fluorescent protein (GFP). (D) Determination of interference efficiency by laser confocal microscopy (200×), DCMECs were transfected with a FAM negative control for 48 h. Nucleusi were stained with DAPI.
(TIF)

Figure S4 **CASY-TT analysis demonstrated viability of DCMECs following transfection.** Viable cells are to the right of the red line, all other cells were non-viable. (A) Non-treated group. DCMECs were non-transfected and cultured for 36 h. (B) Empty vector control group. DCMECs were transfected with pGCMV-IRES-EGFP for 36 h. (C) *Pten* overexpression group. DCMECs were transfected with pGCMV-*Pten*-IRES-EGFP recombinant plasmid for 36 h. (D) Non-treated group. DCMECs were non-transfected and cultured for 48 h. (E) Negative control group. DCMECs were transfected with negative control interference segment for 48 h. (F) *Pten* siRNA group. DCMECs were transfected with *Pten* siRNA interference segment for 48 h.
(TIF)

Table S1 **Ratio of ingredients in the feed given to dairy cows in our study.**
(DOC)
