# Supplementary figures and images for: Pten Regulates Development and Lactation in the Mammary Glands of Dairy Cows
Source: PLoS One. 2014 Jul 10;9(7):e102118. doi: 10.1371/journal.pone.0102118 (PMC4092105; doi:10.1371/journal.pone.0102118)

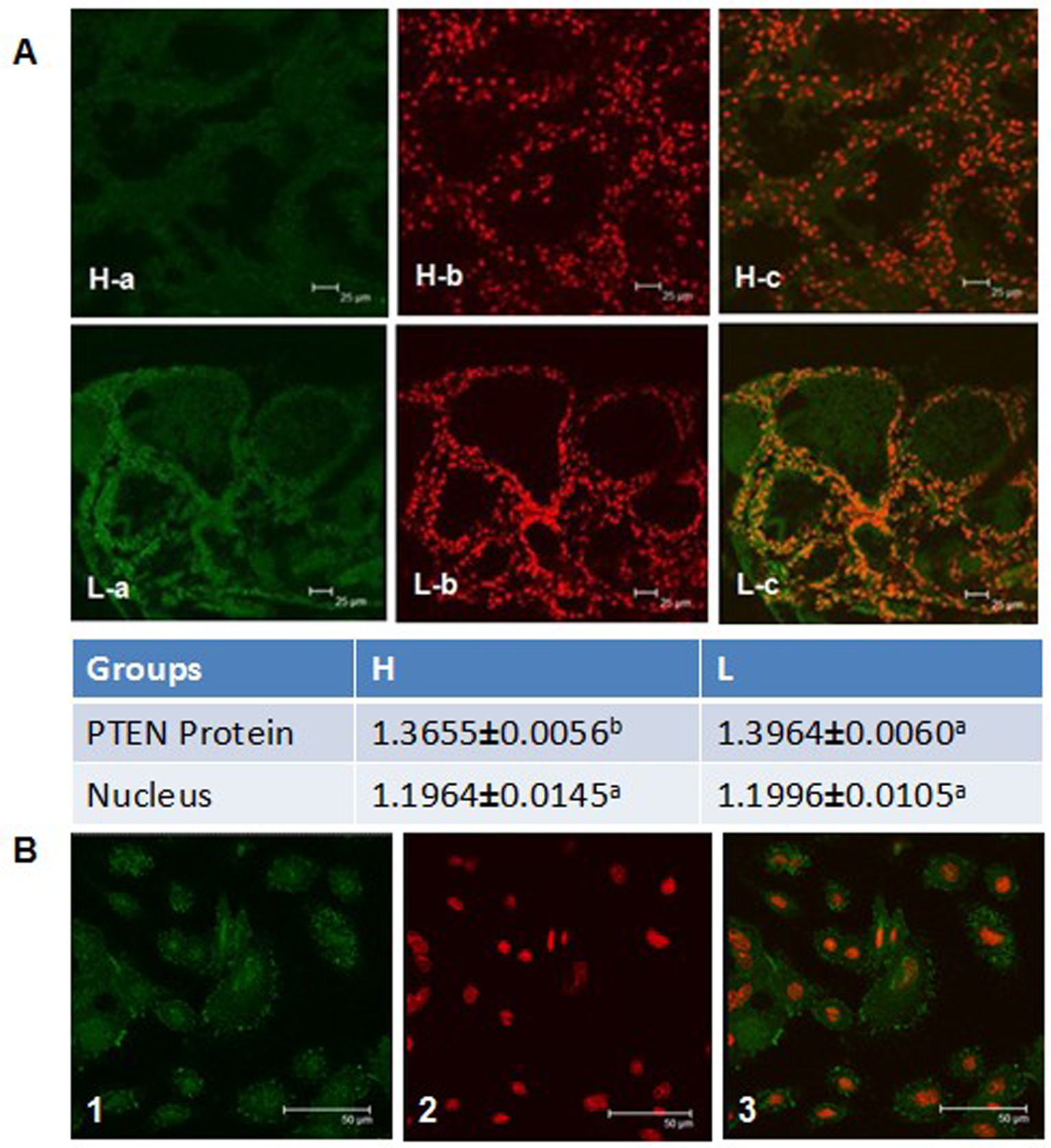

Supplement: Figure S1 — Localization of PTEN in dairy cow mammary tissues and cells. (A) Confocal microscopy images showing localization of PTEN in dairy cow mammary tissues. H, tissue from cows with high quality milk; L, tissue from cows with low quality milk. (a) PTEN, (b) nuclear staining with propidium iodide (PI), (c) merged image of (a) and (b). The mean optical density of PTEN protein expression in different mammary tissues from high or low quality milk producing and lactating dairy cows (n = 3 in each group) are shown in the table below. Each value is presented as the mean ± SD, different superscript letters indicate significantly different values in line data, P<0.05. (B) Localization of PTEN in DCMECs (200×). (1) PTEN; (2) nuclear staining with PI; and (3) merged images of (1) and (2). (TIF) [file pone.0102118.s001.tif]

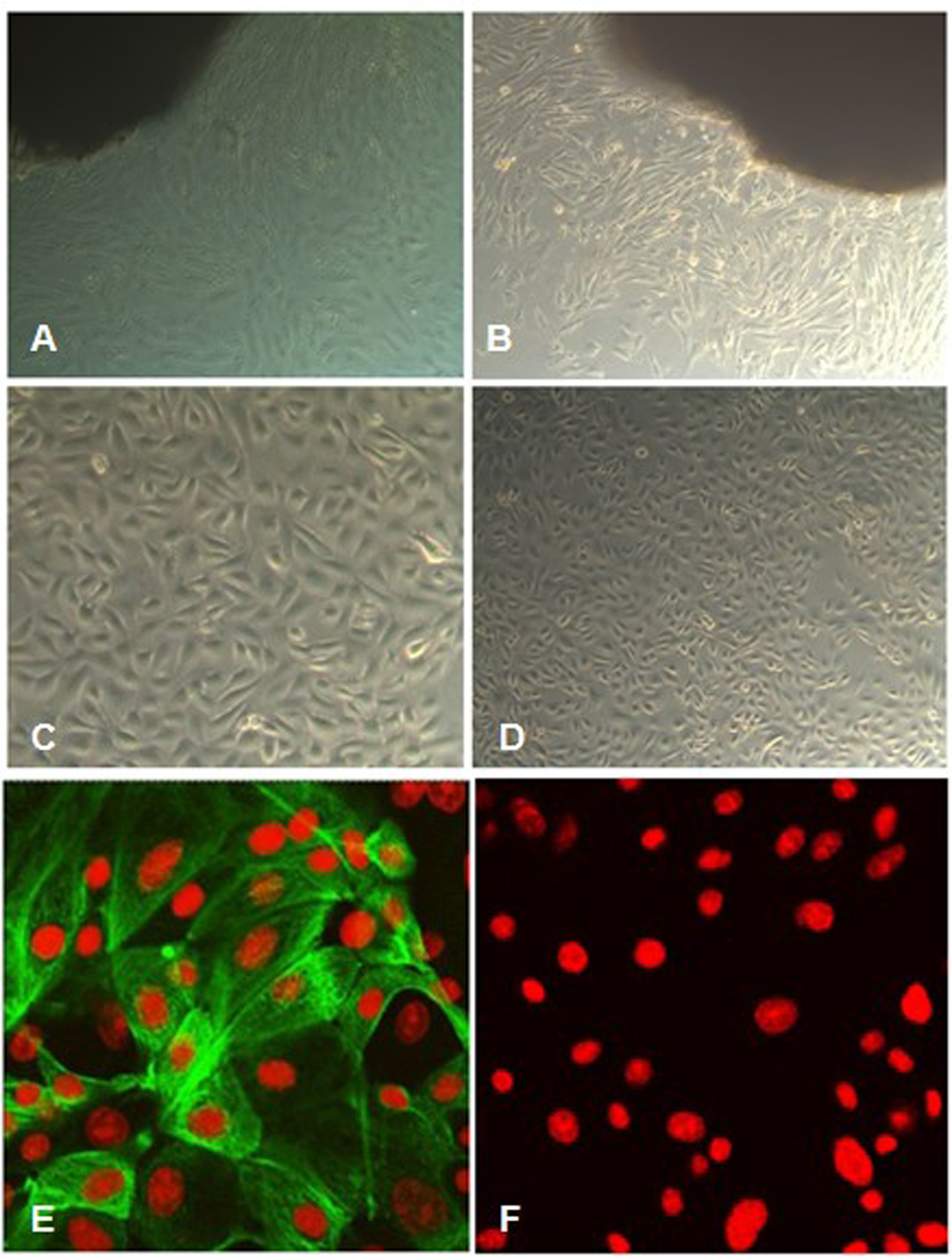

Supplement: Figure S2 — Cultured dairy cow mammary gland epithelial cells. (A) Collagenoblast (200×). (B) Collagenoblast and DCMECs (200×). (C) Purified DCMECs (200×). (D) Serial cultures of DCMECs (200×). (E) Cytokeratin 18 staining of mammary epithelial cells. (F) Cytokeratin 18 staining of fibroblast cells. Nuclei were stained with PI. (TIF) [file pone.0102118.s002.tif]

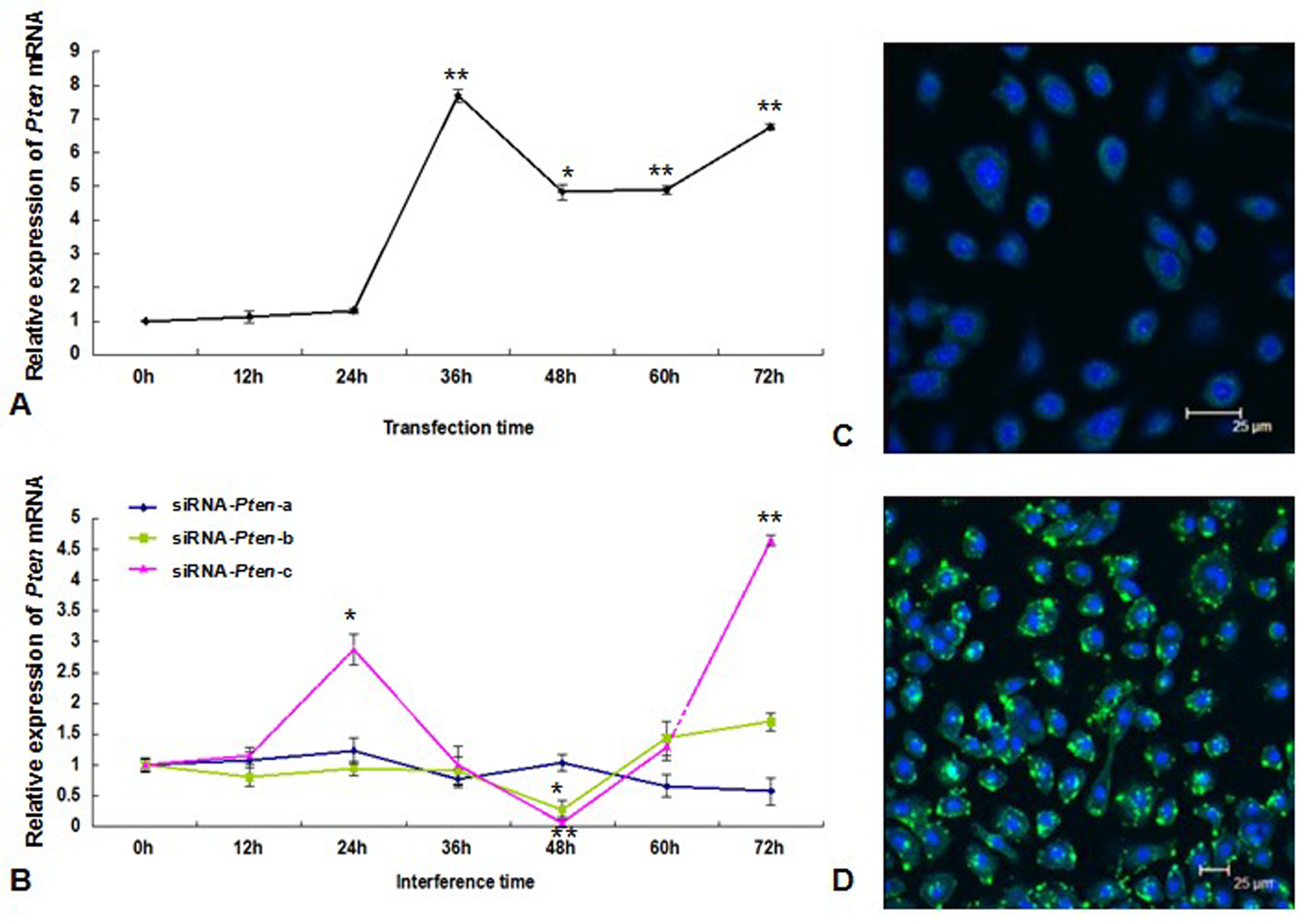

Supplement: Figure S3 — Optimization of transfection conditions. (A) Relative mRNA levels of DCMECs following transfection with Pten recombinant plasmid were determined using qPCR. Expression of Pten mRNA levels peaked at 36 h. Expression was calculated relative to expression levels at 0 h. *P<0.05, **P<0.01. (B) Screening of siRNA efficiency and incubation times. Relative mRNA levels in DCMECs transfected with various siRNAs (siRNA-Pten-a, siRNA-Pten-b and siRNA-Pten-c) at different time points as determined by qPCR. Expression was determined relative to expression levels at 0 h. *P<0.05, **P<0.01. (C) Transfection efficiency as determined by laser confocal microscopy (200×). DCMECs were transfected with the Pten recombinant plasmid for 36 h. Nuclei were stained with DAPI, and PTEN was detected by visualizing green fluorescent protein (GFP). (D) Determination of interference efficiency by laser confocal microscopy (200×), DCMECs were transfected with a FAM negative control for 48 h. Nucleusi were stained with DAPI. (TIF) [file pone.0102118.s003.tif]

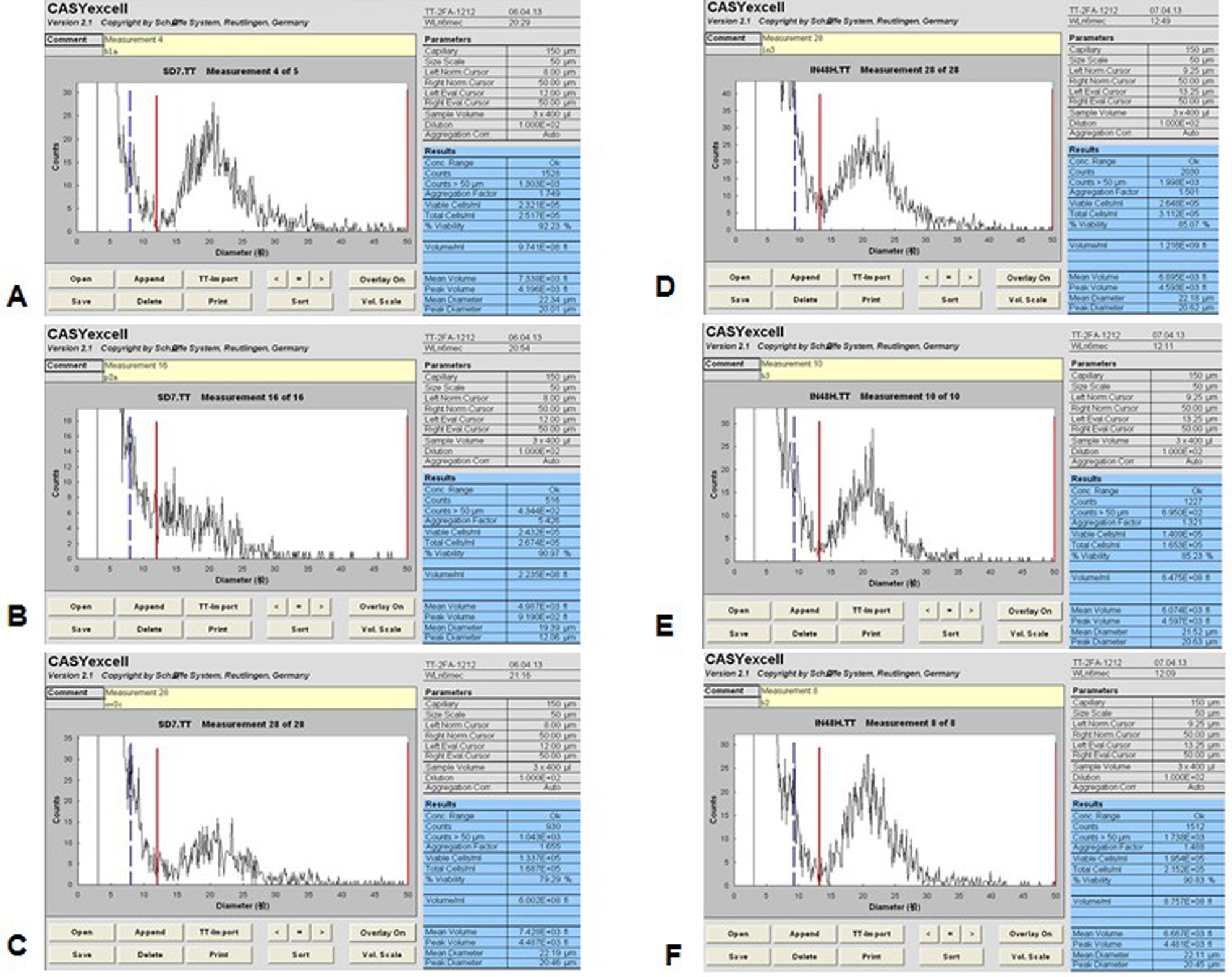

Supplement: Figure S4 — CASY-TT analysis demonstrated viability of DCMECs following transfection. Viable cells are to the right of the red line, all other cells were non-viable. (A) Non-treated group. DCMECs were non-transfected and cultured for 36 h. (B) Empty vector control group. DCMECs were transfected with pGCMV-IRES-EGFP for 36 h. (C) Pten overexpression group. DCMECs were transfected with pGCMV-Pten-IRES-EGFP recombinant plasmid for 36 h. (D) Non-treated group. DCMECs were non-transfected and cultured for 48 h. (E) Negative control group. DCMECs were transfected with negative control interference segment for 48 h. (F) Pten siRNA group. DCMECs were transfected with Pten siRNA interference segment for 48 h. (TIF) [file pone.0102118.s004.tif]
