# Supplementary material for: Pten Regulates Development and Lactation in the Mammary Glands of Dairy Cows
Source: PLoS One. 2014 Jul 10;9(7):e102118. doi: 10.1371/journal.pone.0102118 (PMC4092105; doi:10.1371/journal.pone.0102118)
Supplement: Table S1 — Ratio of ingredients in the feed given to dairy cows in our study. (DOC) [file pone.0102118.s005.doc]

**Table S1. Ratio of ingredients in the feed given to dairy cows in our study**

| Ration composition of 30% fiber diet of the 6 lactating cows | Formulated ingredients (g/kg Dry Matter) |
| --- | --- |
| Wheat silage | 100 |
| Oat hay | 80 |
| Wheat straw |  |
| Sunflower straw |  |
| Corn silage | 100 |
| Clover hay | 23 |
| Soybean hulls | 78 |
| Soybean meal (solvent extracted) | 22 |
| Ground corn grain | 129 |
| Ground barley grain | 87 |
| Ground wheat grain | 44 |
| Whole cottonseeds | 20 |
| Corn gluten Feed | 96 |
| Corn distillers dry grain | 89 |
| Rapeseed meal | 38 |
| Whey solids | 36 |
| NaHCO_3_ | 7.4 |
| NaCl | 6 |
| CaCO_3_ | 9 |
| ^1^Ca-LCFA | 14 |
| Soy molassa | 17 |
| Urea | 4 |
| Trace mineral+vitamin mixture^2^ | 0.6 |

Note: ^1^Calcium salts of long-chain fatty acids; ^2^The trace minerals + vitamins mix contained (g/kg DM): Zn, 24; Fe, 24; Cu, 12.8; Mn, 24; I, 1.44; Co, 0.32; Se, 0.32; Vit. A 16,000,000 IU; Vit. D_3_, 3,200,000 IU; Vitamin E, 48,000 IU.
